# Interprofessional Collaboration between ICU Physicians, Staff Nurses, and Hospital Pharmacists Optimizes Antimicrobial Treatment and Improves Quality of Care and Economic Outcome

**DOI:** 10.3390/antibiotics11030381

**Published:** 2022-03-13

**Authors:** Stephan Schmid, Sophie Schlosser, Karsten Gülow, Vlad Pavel, Martina Müller, Alexander Kratzer

**Affiliations:** 1 Department of Internal Medicine I, Gastroenterology, Hepatology, Endocrinology, Rheumatology, and Infectious Diseases, University Hospital Regensburg, Franz-Josef-Strauß-Allee 11, 93053 Regensburg, Germany; sophie.schlosser@ukr.de (S.S.); karsten.guelow@ukr.de (K.G.); vlad.pavel@ukr.de (V.P.); martina.mueller-schilling@ukr.de (M.M.); 2 Hospital Pharmacy, University Hospital Regensburg, Franz-Josef-Strauß-Allee 11, 93053 Regensburg, Germany; alexander.kratzer@ukr.de

**Keywords:** infectious diseases, intensive care unit (ICU), interprofessional collaboration, carbapenem antibiotics, hospital pharmacists, antimicrobial resistance, antibiotic prescribing

## Abstract

(1) Background: Antibiotic resistance is a worldwide health threat. The WHO published a global strategic plan in 2001 to contain antimicrobial resistance. In the following year, a workshop identified crucial barriers to the implementation of the strategy, e.g., underdeveloped health infrastructures and the scarcity of valid data as well as a lack of implementation of antibiotic stewardship (ABS) programs in medical curricula. Here, we show that interprofessional learning and education can contribute to the optimization of antibiotic use and preserving antibiotic effectiveness. We have initiated interprofessional rounds on a medical intensive care unit (MICU) with a focus on gastroenterology, hepatology, infectious diseases, endocrinology, and liver transplantation. We integrated ICU physicians, hospital pharmacists, nursing staff, and medical students as well as students of pharmacy to broaden the rather technical concept of ABS with an interprofessional approach to conceptualize awareness and behavioral change in antibiotic prescription and use. Methods: Clinical performance data and consumption figures for antibiotics were analyzed over a 10-year period from 2012 to 2021. The control period covered the years 2012–2014. The intervention period comprised the years 2015–2021, following the implementation of an interprofessional approach to ABS at a MICU of a German university hospital. Data from the hospital pharmacy, hospital administration, and hospital information system were included in the analyses. A specific electronic platform was developed for the optimization of documentation, interprofessional learning, education, and sustainability. The years 2020 and 2021 were analyzed independently due to the SARS-CoV-2 pandemic and the care of numerous COVID-19 patients at the MICU. Results: Implementation of an interprofessional ABS program resulted in the optimization of antibiotic management at the MICU. The suggestions of the hospital pharmacist for optimization can be divided into the following categories (i) indication for and selection of therapy (43.6%), (ii) optimization of dosing (27.6%), (iii) drug interactions (9.4%), (iv) side effects (4.1%), and (v) other pharmacokinetic, pharmacodynamic, and pharmacoeconomic topics (15.3%). These suggestions were discussed among the interprofessional team at the MICU; 86.1% were consequently implemented and the prescription of antibiotics was changed. In addition, further analysis of the intensive care German Diagnosis Related Groups (G-DRGs) showed that the case mix points increased significantly by 31.6% during the period under review. Accordingly, the severity of illness of the patients treated at the ICU as measured by the Simplified Acute Physiology Score (SAPS) II increased by 21.4% and the proportion of mechanically ventilated patients exceeded 50%. Antibiotic spending per case mix point was calculated. While spending was EUR 60.22 per case mix point in 2015, this was reduced by 42.9% to EUR 34.37 per case mix point by 2019, following the implementation of the interprofessional ABS program on the MICU. Through close interprofessional collaboration between physicians, hospital pharmacists, and staff nurses, the consumption of broad-spectrum antibiotics, e.g., carbapenems, was significantly reduced, thus improving patient care. In parallel, the case mix and case mix index increased. Thus, the responsible use of resources and high-performance medicine are not contradictory. In our view, close interprofessional and interdisciplinary collaboration between physicians, pharmacists, and nursing staff will be of outstanding importance in the future to prepare health care professionals for global health care to ensure that the effectiveness of our antibiotics is preserved.

## 1. Introduction

Antimicrobial resistance (AMR) is a global threat to human health. AMR is a leading cause of death worldwide with an especially high burden in low-resource countries [1]. According to the World Health Organization (WHO), bacterial antimicrobial resistance is one of the biggest threats to global health, food security, and development. Antibiotic resistance can affect anyone, of any age, in any country, and leads to longer hospital stays, higher medical costs, and increased mortality [2]. Most recent estimates have revealed that, by 2050, antibiotic resistance will have reduced the world’s population by as much as 444 million [3,4]. According to the Antimicrobial Resistance Collaborators [5], AMR is a health problem whose dimensions are at least comparable to major diseases such as HIV and malaria. Furthermore, the Antimicrobial Resistance Collaborators identified six leading pathogens for deaths attributed to resistance, 1. *Escherichia coli,* followed by 2. *Staphylococcus aureus,* 3. *Klebsiella pneumoniae,* 4. *Streptococcus pneumoniae,* 5. *Acinetobacter baumannii,* and 6. *Pseudomonas aeruginosa.*

Antibiotic resistance is of particular relevance for intensive care medicine, as ICU physicians worldwide are facing more and more patients infected by bacteria for which limited or no adequate therapeutic options are available [6,7]. In intensive care units (ICUs), incidence rates for vancomycin-resistant enterococci (VRE) and Enterobacteriaceae resistant to third-generation cephalosporins and fluoroquinolones have been increasing [8]. Of particular concern is the increase in carbapenem-resistant *K. pneumoniae* [9].

It has been shown that the use of antibiotics in ICUs, especially of carbapenems, is associated with the increasing development of antibiotic resistance [10]. The WHO and multiple other groups and researchers agree that the increase in AMR requires global action and a strategic plan. A “One Health” approach is needed here; because AMR is such an essential global issue, to combat AMR, multiple sectors need to communicate and work together to achieve better public health outcomes [4,11,12,13,14]. Therefore, as early as 2011, the WHO published a European strategic action plan on antibiotic resistance [15]. This action plan has been implemented in Germany through the S3 Guideline “Strategies for ensuring rational antibiotic use”, which identifies the requirements and core strategies for antibiotic stewardship [16,17].

One of the core elements of antibiotic stewardship is the cooperation between ICU physicians and hospital pharmacists with special qualifications in infectious diseases. We aimed to further optimize antibiotic stewardship (ABS) and integrate ABS into intensive care interprofessional grand rounds with attending ICU physicians, hospital pharmacists, and ICU nursing staff. We aimed to facilitate and initiate interprofessional ABS, integrating and empowering physicians, hospital pharmacists, and nurses. Furthermore, we added ABS education to the specific in-house curricula for students of medicine, pharmacy, and nursing [18]. To adequately educate healthcare professionals and students for a global society, we suggest expanding and optimizing interprofessional ABS [19,20,21,22].

Our MICU at a German university medical center is specialized in the treatment of gastrointestinal and liver diseases. Particularly noteworthy is the MICU’s focus on liver transplantation, which is reflected in the high number of patients on the waiting list for liver transplantation with acute decompensation of disease and acute-on-chronic liver failure. Infections in patients with cirrhosis represent an increasing health and economic burden [23,24]. Patients with liver cirrhosis are prone to acute decompensation, acute-on-chronic liver failure (ACLF), and hospitalization, including the application of broad-spectrum antibiotics due to infections. Infections constitute the most frequent etiology of ACLF which is defined by the presence of organ failure [25,26,27]. Therefore, infections have been proposed as the fourth major complication of liver cirrhosis in addition to ascites, hepatic encephalopathy, and gastrointestinal hemorrhage [28]. Among bacterial infections, spontaneous bacterial peritonitis, sepsis, and pneumonia were more frequently associated with ACLF than other infections in the CANONIC study [27].

Of major concern is the increasing prevalence of infections with multidrug-resistant organisms in patients with chronic liver disease [29,30,31,32]. Thus, patients on the waiting list for liver transplantation constitute a particularly vulnerable patient group, and implementation of infection control measures and optimized ABS programs are essential in transplantation centers. 

Here, we have addressed the following topics among the broad spectrum of ABS: (i) indication and selection of therapy, (ii) optimization of dosing, (iii) drug interactions, (iv) side effects, and (v) pharmacokinetic, pharmacodynamic, and pharmacoeconomic issues. To combat AMR, a “One Health” strategy is needed. This strategy relies on interprofessional learning and education to develop healthcare professionals who have learned to cooperate across and beyond disciplines to continuously improve the quality of care. Here, we show our results after the implementation of an interprofessional approach to ABS. Taken together, the knowledge and skills of healthcare professionals in prescribing antimicrobial drugs improved. Furthermore, carbapenem consumption decreased as an important result of our tailored and interprofessional approach to ABS. In contrast to the increasing numbers of critically ill patients and the augmenting severity of diseases, expenditure, and cost of antibiotics on our medical intensive care unit (MICU) declined. This highlights the importance and the benefit of continuous monitoring of antibiotic consumption in (M) ICUs.

## 2. Results

In this analysis, the medical and economic results of an interprofessional approach to ABS through comprehensive collaboration between ICU physicians, pharmacists, and nursing staff at a MICU were studied over a 10-year period from 2012 until 2021. The control period comprised the years 2012–2014. In 2015 we started our interprofessional ABS intervention including interprofessional training of intensive care physicians, hospital pharmacists, medical students, pharmacology students, nurses, and clinical nurse assistants. Our aim was to improve knowledge about antibiotics and to strengthen antimicrobial optimization in the ICU.

Our MICU at a German university medical center has a specific focus on gastroenterology, hepatology, infectious diseases, and endocrinology. Particularly noteworthy is the MICU’s focus on transplant medicine, which is reflected in the high number of immunosuppressed patients. The unit is comprised of 14 full-service intensive care beds under the guidance of a gastroenterologist and a multidisciplinary critical care team composed of physicians, nurses, respiratory therapists, occupational therapists, speech therapists, pharmacists, case coordinators, and medical and pharmacology students. Patients were often transferred from other hospitals for liver transplantation or interventional endoscopy.

As the patient population on our MICU changed from 2020 due to the COVID-19 pandemic, medical and economic results for 2020 and 2021 are presented as separate items.

### 2.1. Establishment of a Close Interprofessional Cooperation between MICU Physicians, Nursing Staff, and Pharmacists

From 2015, i.e., the beginning of the ABS intervention period, we have put a special focus on the close cooperation between the MICU physicians and the pharmacists of the on-site hospital pharmacy. Several pharmacists of the on-site hospital pharmacy are specialized in antimicrobial stewardship. This central axis of cooperation between MICU physicians and pharmacists has continuously been expanded over the years. Furthermore, colleagues from microbiology, virology, and hospital hygiene are involved in ABS in accordance with the national guidelines [16].

There is a very close daily exchange and consultation between the MICU physicians and pharmacists. The pharmacists can assess the medication of all the patients at the MICU at any time by accessing an electronic chart. Thus, the attending MICU physician can consult with the pharmacist at any time. A hospital pharmacist is permanently assigned to the intensive care unit so that there is continuity, and the pharmacist knows the patients in the intensive care unit. Every week, there is a grand round with intensive care physicians, hospital pharmacists, nurses, students, and staff doctors from hospital hygiene, virology, and microbiology. These antibiotic stewardship rounds in our MICU include the rapid identification and optimal treatment of bacterial infections in our critically ill patients, based on pharmacokinetic and pharmacodynamic characteristics, and shortening the duration of antibiotic administration. Furthermore, we aimed to prepare future pharmacists, medical doctors, and nurses for a more comprehensive and interprofessional ABS. We believe that every effort should be made to incorporate interprofessional collaboration into ABS education. In addition, we encouraged the participation of inpatient staff nurses as antimicrobial stewards. Guidelines on antimicrobial stewardship emphasize the importance of an interdisciplinary team, but current practice focuses primarily on defining the role of infectious disease physicians and pharmacists. Therefore, we believe that the role of inpatient staff nurses as antimicrobial stewards should be explored.

### 2.2. Documentation and Implementation of Intervention Proposals

To document the results of an interprofessional approach to ABS and the close interprofessional cooperation between MICU physicians and pharmacists, in March 2018 a joint platform for documenting the corresponding measures was created in the patient data management system (Metavision^®^ System; iMDsoft^®^). 

Based on 742 medication reviews, analyses of the documentation showed that the preparation time of the pharmacists per MICU patient was 19.7 minutes on average. Per medication check, 0.85 intervention proposals were developed by the pharmacist.

**Figure 1:**
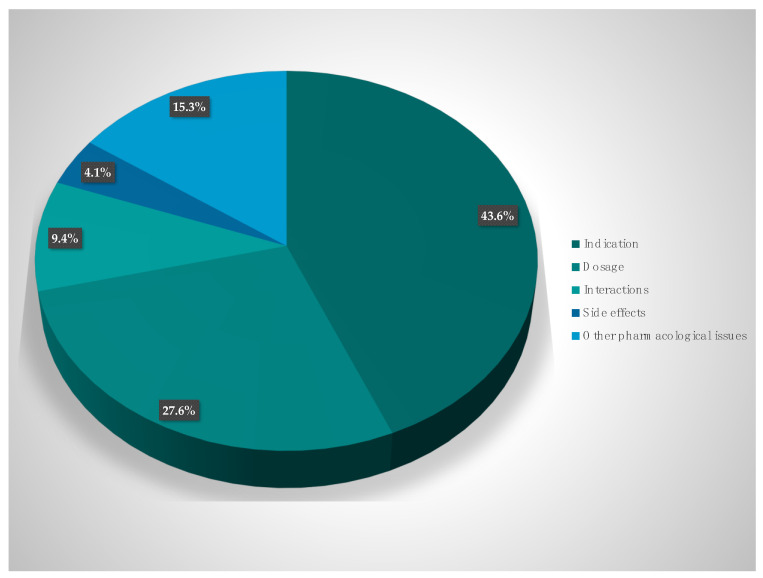
Intervention proposals (%).

The suggestions of the hospital pharmacists for optimization can be divided into the following categories (i) indication and selection of therapy (43.6%), (ii) optimization of dosing (27.6%), (iii) drug interactions (9.4%), (iv) side effects (4.1%), and (v) other pharmacokinetic and pharmacodynamic issues (15.3%). The suggestions of the pharmacists were discussed among the interprofessional team, and 86.1% were consequently implemented and the prescription of antibiotics was changed (Figure 1).

### 2.3. Development of Antibiotic Consumption

Our interprofessional approach to ABS has significantly reduced the use of antibiotics at the MICU, which is shown by a decrease in the use density of antibiotics. Application density is calculated in recommended daily doses (RDD) per 100 patient days (PD), which is an established measurement of hospital antibiotic use [33]. Comparing the years 2015 and 2019, the application density of antibiotics was reduced by 12.2% from 150.9 RDD/100 PD to 132.5 RDD/100 PD.

A particular aim was to review the available data on carbapenem use in our MICU. Carbapenem is a broad-spectrum antibiotic family that keeps an excellent activity to extended-spectrum β-lactamases. It becomes a drug of choice for empirical therapy of suspected sepsis in known or presumably known Extended-spectrum beta-lactamase (ESBL) carriers. However, emerging carbapenem resistance has been related to the increase of carbapenem consumption in ICUs. In contrast, and as a result of our interprofessional ABS, the application density of carbapenems was reduced by 23.4% from 41.1 RDD/100 PD to 31.5 RDD/100 PD (2015 vs. 2019). Comparing 2015 and 2019, the consumption of cephalosporins, fluoroquinolones, glycopeptides, and linezolid were also reduced by 40.0%, 36.9%, 26.4%, and 3.0%, respectively.

**Figure 2:**
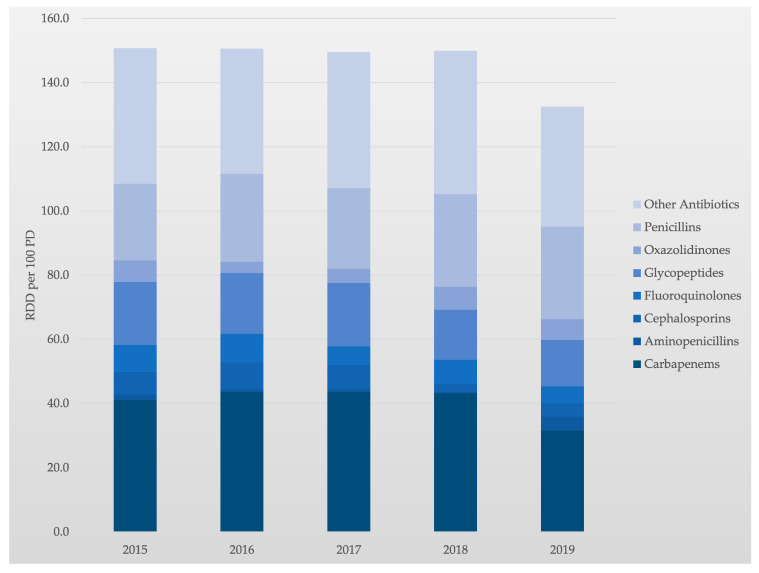
Application density of antibiotics in recommended daily doses (RDD) per 100 patient days (PD).

In parallel, the use of penicillins and aminopenicillins in our MICU increased, by 20.5% and 152.9%, respectively (2015 vs. 2019). This development is due to a change in the antibiotic class away from carbapenems towards penicillins and aminopenicillins. Thus, tailored and interprofessional stewardship programs are essential to better control carbapenem use and subsequent antimicrobial resistance. The development of the application density of antibiotics over time is shown in Table 1 and Figure 2.

### 2.4. Development of Expenditure on Antibiotics

**Figure 3:**
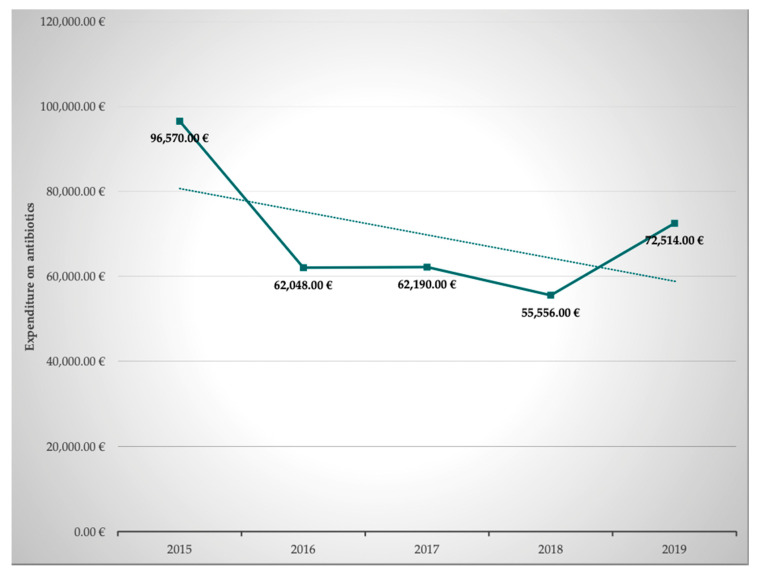
Development of expenditure on antibiotics from 2015 to 2019 in Euro (€).

In addition to the optimization of prescription, dosing, and pharmacokinetic and pharmacodynamic aspects, we set a special focus on pharmacoeconomics. Pharmacoeconomics is a subdiscipline of health economics and evaluates the cost and effects of pharmaceutical drugs or drug therapies. We reasoned that these are important aspects for the curricula of medical doctors, pharmacists, and nursing staff as well. Both inappropriate use of antibiotics and lack of access to antibiotics are threats to global public health and interprofessional education should raise awareness towards socioeconomic as well as sociodemographic data and challenges. The corresponding data of our MICU were obtained from our hospital pharmacists. The total overall expenditure on antibiotics per year clearly decreased by 24.9% from EUR 96,570.75 to EUR 72,514.54 in the observed 5-year period between 2015 and 2019. An overview of the development of the expenditure on antibiotics is given in Table 2 and Figure 3.

### 2.5. Development of Clinical Performance

The clinical performance indicators of the MICU were comprehensively analyzed. Therefore, occupancy rate (in%), length of stay (in days), mechanical ventilation (in%), and Simplified Acute Physiological Score II (SAPS II)/bed and case mix points (total, per year, German-Diagnosis Related Groups (G-DRGs)) were included in our analyzes.

The occupancy rate was consistently at a high level, averaging 89.9%, reflecting the permanently high demand for beds at this specific (liver and infectious diseases and liver transplant) MICU. Due to the high severity of their disease, the length of stay of the patients was 8.3 days on average. Overall, there was a slight decrease in the length of stay between 2015 and 2019. On average, 50.8% of patients were mechanically ventilated. The ventilation rate increased by 8.7% to 51.3% in the analyzed period.

The severity of the disease is particularly well represented by the SAPS II, which was automatically calculated and documented daily at midnight by the patient data management system (Metavision^®^ System; iMDsoft^®^). On average, 15,548 SAPS II points per bed and per year were achieved in the MICU. Comparing the years 2015 and 2019, the SAPS score increased by 21.4%.

By analyzing the intensive care G-DRGs obtained in the department, the case mix points generated in the ICU were calculated. On average, 1915 ± 239 case mix points were generated at the MICU per year. Comparing the years 2015 and 2019, case mix points increased by 31.6% from 1603 to 2109. Furthermore, it must be taken into consideration that additional interventional procedures have been performed in the MICU which also triggered non-intensive care G-DRGs and were included in the department’s total G-DRG reimbursement. Thus, the case mix points achieved in the MICU are higher than those shown in Table 3.

**Figure 4:**
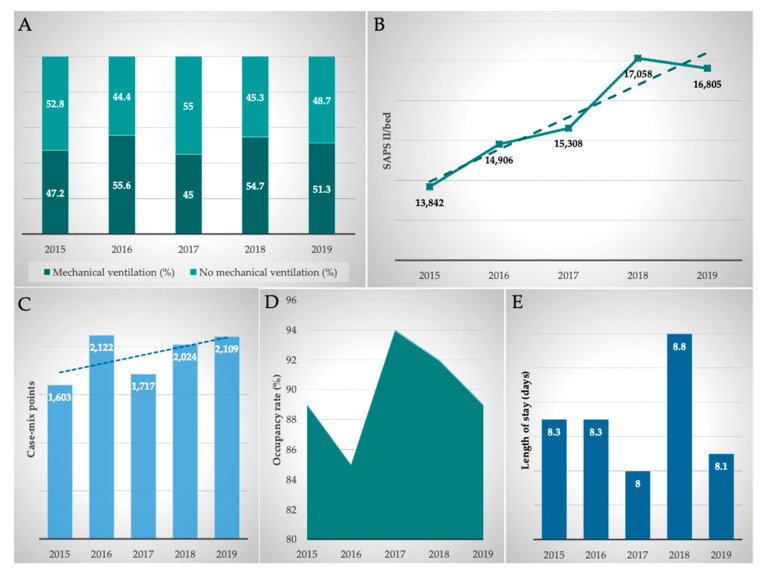
Development of clinical performance from 2015 to 2019. (**A**) Mechanical ventilation; (**B**) SAPS II/bed, SAPS = Simplified Acute Physiology Score; (**C**) Case mix points; (**D**) = Occupancy rate (%); and (**E**) = Length of stay (days).

In summary, all parameters examined showed a good development of clinical performance, with the rise in SAPS II points (+21.4%) and case mix points (+31.6%) being particularly noteworthy. An overview of clinical performance parameters is given in Figure 4.

### 2.6. Calculation of the Expenditure on Antibiotics per Case Mix Point–G-DRG

Subsequently, the annual development of expenditure on antibiotics per case mix point was calculated based on the costs for antibiotics and the achieved case mix points. This revealed that the expenditure on antibiotics per case mix point averaged 37.50 ± 13.20 €, decreasing from 60.22 € in 2015 to 34.37 € in 2019. This represents savings of 41.9%. In parallel and as shown above, application density (RDD/100 PD) of antibiotics decreased. Thus, the decline in expenditure on antibiotics is clearly an effect of interprofessional ABS and due to less antibiotic use, not due to lower prices of antibiotics (Table 1, Figure 2).

**Figure 5:**
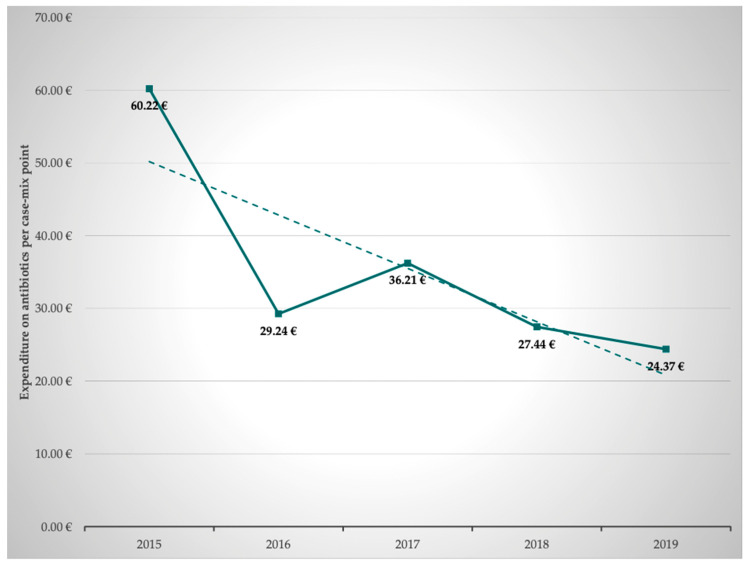
Expenditure on antibiotics per case mix point.

The development of the expenditure on antibiotics per case mix point is shown in Table 4 and Figure 5. Based on the respective G-DRG state basal rates, we could show that 1.85% (2015) and 0.97% (2019) of the revenue generated by the G-DRGs was spent on antibiotics. This calculation shows a 52% reduction in expenditure on antibiotics.

### 2.7. Comparison with Control Period (2012–2014) before Implementation of Interprofessional ABS

**Figure 6:**
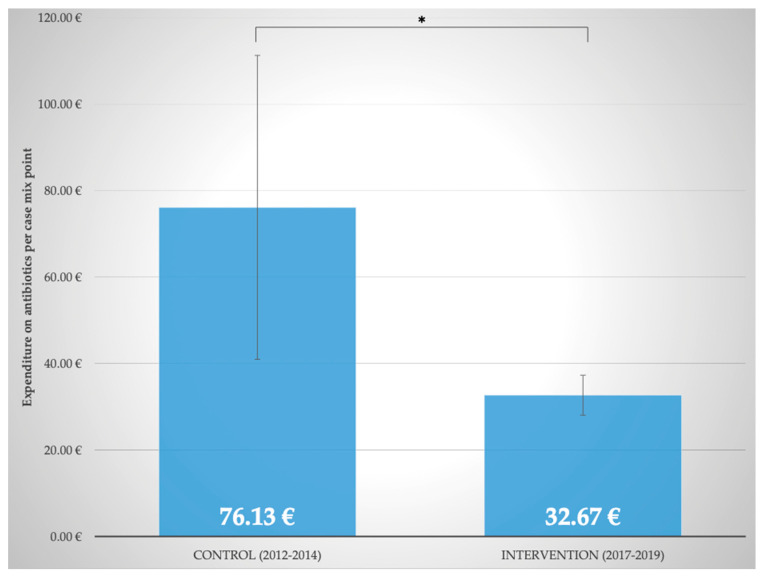
Expenditure on antibiotics per case mix point (€) in the control (2012–2014) and intervention group (2017–2019); * *p* = 0.032.

To further analyze the benefits of the close interprofessional cooperation between physicians, pharmacists, and nurses in ABS, a comparison with a control period from 2012–2014 was performed. As displayed in Figure 6, expenditure on antibiotics per case mix point significantly decreased (*p* ≤ 0.05) from 76.13 ± 35.19 EUR (2012–2014, control period) to 32.67 ± 4.62 EUR (2017–2019, last three years of intervention period). Moreover, despite an increase in disease severity, isolations of patients due to multidrug-resistant pathogens were significantly (*p* ≤ 0.05) reduced from 40.81 ± 3.84% to 27.00 ± 2.78% (2012–2014, control period vs. 2017–2019, last three years of intervention period). This is an indication that infectious complications decreased.

### 2.8. Developments during the SARS-CoV-2 Pandemic

Due to structures that had been well established for years, the close interprofessional collaboration between MICU physicians and pharmacists was continued and intensified during the SARS-CoV-2 pandemic. This was an important support in the care of the often complex and seriously ill COVID-19 patients.

During the SARS-CoV-2 pandemic in 2020, there was a surge in the consumption density of antibiotics to 155.4 RDD/100 PD and an increase in total expenditure on antibiotics to 76,764 EUR. This increase can be explained by the numbers of severely ill COVID-19 patients treated in the ward, who often required long and comprehensive antibiotic therapy due to bacterial superinfections. Furthermore, the SARS-CoV-2 pandemic also led to price increases for some antibiotics. However, in 2021, despite continued treatment of COVID-19 patients, consumption density reduced to 147.8 RDD/100 PD and the expenditures declined to 75,292 EUR.

## 3. Discussion

Here, we suggest extending the concept of ABS beyond the technical aspects of antibiotic resistance to a health systems approach to preserve antibiotic effectiveness as a “One Health” strategy. The focus of our study was to implement and evaluate the impact of interprofessional education, learning, and collaboration of hospital pharmacists, attending ICU physicians, and ICU nursing staff in a comprehensive approach to ABS on the application density of antibiotics, quality of care, and expenditure in a MICU of a university center of tertiary care.

Our special focus was on the interprofessional ABS education of health care professionals and students to encourage cooperation across disciplines and professions.

Our results show that over a 7-year intervention period, despite increasing case severity of our patients, fewer antibiotics, and especially fewer broad-spectrum antibiotics, e.g., carbapenems, were used. In addition, overall expenditure on antibiotics decreased. From our point of view, the interprofessional collaboration led to an enhanced understanding of the “silent pandemic” [34] of antibiotic resistance and how it can be addressed from a health systems perspective beyond disciplines and professions. The value of the hospital pharmacist in antibiotic stewardship has been highlighted before [19,35,36,37]. Evidence on the formal inclusion of nurses in ABS remains limited. Formal inclusion of nurses in ABS activities has been associated with improved nurse knowledge, nurse confidence, and improved clinical outcomes for patients [38]. However, the formal inclusion of nurses in ABS does not yet represent actual clinical practice. Our department has a strong focus on interprofessional learning and education. Consequently, we have implemented ABS on the MICU, integrating staff nurses and nurses at the bedside. We think that there is an urgent need for interprofessional cooperation to support a health systems approach to contain antimicrobial resistance. Therefore, and in addition to the MICU rounds, we have created a new online platform that enables not only documentation but also communication and sustainability.

The first step in the development of any interprofessional antibiotic stewardship (ABS) program is to build an interprofessional and multidisciplinary team encompassing the necessary expertise in the complex management of infections and interprofessional learning. Interprofessional education is an approach recommended for improving the prescribing practice of antibiotics [21,39,40]. We cannot exclude that—in addition to the implementation of our interprofessional ABS program—other practice changes, for example, better use of VAP prevention or implementation of new ICU guidelines contributed to the observed positive effects on antibiotic application density, expenditure, and quality of care. However, there was no change neither in the key individuals of the MICU team nor in the structure of our MICU during the observation period that may have confounded the outcome of our study.

The benefit of interprofessional ABS outweighs the time invested by the health professionals. The pharmacist’s weekly preparation time averaged 19.7 min per patient. In the current literature, 30 min per patient has been suggested for preparation [37]. The difference between our data and the literature may be explained by the fact that a specific pharmacist is permanently assigned to our MICU and thus knows the patients and their diseases and clinical course very well.

The suggestions by the interprofessional team can be grouped into the following categories: (i) indication and selection of therapy (43.6%), (ii) optimization of dosing (27.6%), (iii) drug interactions (9.4%), (iv) side effects (4.1%), and (v) pharmacokinetic, pharmacodynamic and pharmacoeconomic issues (15.3%). This is in accordance with the current literature which reports on dose adjustments and specific therapeutic indications as the most frequent recommendations [41] resulting from ABS. A total of 86.1% of the recommendations of the hospital pharmacist were fully implemented. This very good implementation rate is most likely due to the interprofessional communication between ICU doctors, ICU nurses, and pharmacists. Previous studies reported implementation rates of only 50% when the ABS consultation was restricted to a written note only [42,43].

In our study, the overall consumption of antibiotics was reduced by 12.2% from 150.9 RDD/100 PD to 132.5 RDD/100 PD in the years 2015 to 2019. At first glance, this appears to be less than in comparable studies, which reported a reduction in the consumption density of antibiotics of approximately 20% [8,44]. When interpreting this data, it must be taken into consideration that the clinical performance of our MICU in this period increased significantly. This is reflected by the increase in SAPS II/bed by 21.4% and the increase in case mix points by 31.6%. In addition, the expenditure on antibiotics was analyzed, showing a decrease of 24.9%. This observation is in line with the literature [41]. Finally, the expenditure on antibiotics was set in relation to the case mix points. This revealed a marked decrease in the antibiotic expenditure of 42.9% per case mix point. From our point of view, this ratio best reflects the savings achieved through the interprofessional collaboration between ICU doctors, nurses, and pharmacists while treating patients with increasing severity of their disease over the years as a tertiary referral center.

Not only did we succeed in reducing the overall consumption density and expenditure of antibiotics, but we also paid special attention to reducing broad-spectrum antibiotics, e.g., carbapenems. In the observation period, the application density of carbapenems reduced 23.4% from 41.1 RDD/100 PD to 31.5 RDD/100 PD (2015 vs. 2019). The reduction of the consumption density of carbapenems is considered a generally accepted and “read out” goal of ABS and is clearly substantiated in the corresponding guidelines. Of clinical importance, not only is the reduction of the consumption of carbapenems a declared goal of ABS but also a reduction in the consumption of other broad-spectrum antibiotic groups should be achieved [16]. We accomplished a decline in the consumption of cephalosporins, fluoroquinolones, glycopeptides, and linezolid by 40.0%, 36.9%, 26.4%, and 3.0%, respectively, following the implementation of interprofessional ABS on the MICU.

Since penicillins and aminopenicillins are considered ideal in the context of antimicrobial resistance, it is a declared goal to switch from other antibiotic classes to aminopenicillins if possible. Overall, the use of penicillins and aminopenicillins has increased in German intensive care units in recent years. In our analysis, the use of penicillins and aminopenicillins clearly increased, by 20.5% and 152.9%, respectively (2015 vs. 2019).

In summary, it can be shown that comprehensive interprofessional cooperation between ICU physicians, ICU nurses, and hospital pharmacists in a comprehensive approach to ABS can achieve a clear reduction in the consumption density of antibiotics. Furthermore, a switch towards penicillins and aminopenicillins can clearly be achieved in severely ill MICU patients. The benefits for patient care come with economic benefits and thus represents multiple wins.

Based on our data, we suggest interprofessional cooperation among ICU physicians, ICU nurses, and hospital pharmacists as an innovative and sustainable approach to optimize future ABS programs and to educate health care professionals for a global health systems approach.

## 4. Materials and Methods

The present study is a retrospective analysis conducted at the MICU of the Department of Internal Medicine I at the University Hospital Regensburg. The MICU is specialized in gastroenterology, hepatology, infectious diseases, endocrinology, rheumatology, and liver transplantation. On average, 14 beds have been operated during the study period. The ICU’s catchment area includes 2.0 million people from the south of Germany; it provides tertiary clinical care and tertiary referral-center care functions.

Primary data were obtained from the SAP^®^ (Systemanalyse Programmentwicklung, Walldorf, Germany) hospital system and the Metavision^®^ patient data management system. In addition, pharmacoeconomic data were provided by the hospital pharmacists and financial reports from the hospital administration. Statistical analyses were performed with the help of SPSS^®^ (Statistical Package for Social Sciences, IBM, Armonk, New York, United States). A one-tailed t-test was performed; *p*-values less than or equal to 0.05 were considered statistically significant. This study was granted approval by the ethics committee of the University Hospital Regensburg, Regensburg, Germany (21-2520-104, 14 July 2021).

In this study, a 10-year period between 2012 and 2021 was analyzed. The control period comprised the years 2012–2014 and the intervention period was from 2015 to 2021. The years 2020 and 2021 were examined separately due to the extraordinary situation caused by the COVID-19 pandemic and the resulting change in the patient collective on the MICU.

Of clinical importance, a joint platform in the patient data management system (Metavision^®^, iMDsoft^®^, Düsseldorf, Germany) was created for the systematic documentation of the ABS-related therapy adjustments which were categorized in antibiotic use before and after the interprofessional grand round and included specific ABS strategies like de-escalation, duration of treatment, and administration optimization. A specific focus was set on the reduction in the prescribing of broad-spectrum antibiotics.

Of clinical relevance, critical illness is associated with changes in pharmacokinetics and pharmacodynamics, which challenge dose-finding and optimization. This is particularly relevant for hydrophilic drugs, e.g., beta-lactam antibiotics. Therefore, special attention was paid to exploring these challenges influencing optimal antibiotic application in the intensive care setting.

Furthermore, the engagement of bedside nurses in antimicrobial stewardship and infection prevention activities was encouraged. We believe that novel strategies to integrate bedside nurses in antimicrobial stewardship are needed and that the respective curricula will have to be adapted accordingly. In addition, we are dedicated to improving interprofessional education on antimicrobial stewardship of all the professions represented in our grand rounds and the application of antibiotics in ICUs.

The application density of antibiotics is presented in recommended daily dose per 100 patient days (RDD/100 PD), which is a reliable benchmark for assessing trends in prescriptions. In our analysis, RDD was used as a scale for the use of antibiotics as it shows smaller deviations from actual prescriptions compared with the WHO-DDD (Defined Daily Dose) system, which may lead to misclassifications in benchmark analyses [33].

## 5. Conclusions

Through interprofessional collaboration between physicians, hospital pharmacists, and nurses, we could broaden the concept of ABS and integrate interprofessional education and interprofessional learning to better understand antibiotic resistance and to optimize the use of antibiotics in the ICU. In our study, an interprofessional approach to ABS led to a decrease in overall antibiotic consumption, a marked decline in the prescription of broad-spectrum antibiotics, and achieved better economic results.

Thus, the responsible use of resources and high-performance medicine are not contradictory. In our view, close interprofessional and interdisciplinary collaboration will be of outstanding importance in the future for the implementation of global strategies to contain antimicrobial resistance and to initiate a health systems approach to contain antibiotic resistance.

## Figures and Tables

**Table 1:** Application density of antibiotics in RDD per 100 PD.

	2015	2016	2017	2018	2019	Mean ± SD	Δ 2019 vs. 2015 (%)
Aminopenicillins	1.7	0.8	0.9	0.4	4.3	1.6 ± 1.6	152.9%
Carbapenems	41.1	43.6	43.5	43.2	31.5	40.6 ± 5.2	−23.4%
Cephalosporins	7.0	8.4	7.6	2.5	4.2	5.9 ± 2.5	−40.0%
Fluoroquinolones	8.4	8.9	5.8	7.5	5.3	7.2 ± 1.6	−36.9%
Glycopeptides	19.7	19.0	19.8	15.6	14.5	17.7 ± 2.5	−26.4%
Oxazolidinones	6.7	3.5	4.4	7.2	6.5	5.7 ± 1.6	−3.0%
Penicillins	23.9	27.4	25.1	28.8	28.8	26.8 ± 2.2	20.5%
All Antibiotics	150.9	150.6	149.5	149.9	132.5	146.68 ± 7.9	−12.2%

Development of consumption density of antibiotics by classes of antibiotics from 2015 to 2019. Mean ± SD and Δ 2019 vs. 2015. RDD = recommended daily doses, PD = patient days.

**Table 2:** Expenditure on antibiotics.

2015	2016	2017	2018	2019	Mean ± SD	Δ 2019 vs. 2015 (%)
96,570 €	62,048 €	62,190 €	55,556 €	72,514 €	71,817 ± 13,650 €	−24.9%

Development of expenditure on antibiotics from 2015 to 2019 in Euro (€). Mean ± SD and Δ2019 vs. 2015.

**Table 3:** Clinical performance.

	2015	2016	2017	2018	2019	Mean ± SD	Δ 2019 vs. 2015 (%)
Occupancy rate (%)	89.0	85.0	94.0	92.0	89.0	89.9 ± 3.4	0.0%
Length of stay (days)	8.3	8.3	8.0	8.8	8.1	8.3 ± 0.3	−2.0%
Mechanical ventilation (%)	47.2	55.6	45.0	54.7	51.3	50.8 ± 4.6	8.7%
SAPS II/bed	13,842	14,906	15,308	17,058	16,805	15,548 ± 1344	21.4%
Case mix points	1603	2122	1717	2024	2109	1915 ± 239	31.6%

Development of clinical performance from 2015 to 2019. Mean ± SD and Δ 2019 vs. 2015. SAPS = Simplified Acute Physiology Score.

**Table 4:** Spending on antibiotics per case mix point.

2015	2016	2017	2018	2019	Mean ± SD	Δ 2019 vs. 2015 (%)
60.22 €	29.24 €	36.21 €	27.44 €	34.37 €	37.50 ± 13.20 €	−42.9%

Development of expenditure on antibiotics per case mix point from 2015 to 2019. Mean ± SD and Δ2019 vs. 2015.

## Data Availability

The data presented in this study are available on request from the corresponding author. The data are not publicly available due to privacy.
